# Genome Sequence of the Relapsing Fever Borreliosis Species *Borrelia hispanica*

**DOI:** 10.1128/genomeA.01171-13

**Published:** 2014-01-16

**Authors:** Haitham Elbir, Pär Larsson, Mukunda Upreti, Johan Normark, Sven Bergström

**Affiliations:** Department of Molecular Biology, Umeå University, and Laboratory for Molecular Infection Medicine Sweden (MIMS), Umeå Centre for Microbial Research (UCMR), Umeå University, Umeå, Swedena; Division of CBRN Security and Defence, FOI Swedish Defence Research Agency, Umeå, Swedenb

## Abstract

*Borrelia hispanica* is the etiological pathogen of tick-borne relapsing fever, transmitted to humans by infected *Ornithodoros erraticus* ticks. Here we present the 1,783,846-bp draft genome sequence, with an average G+C content of 28%. It has 2,140 open reading frames, 3 ribosomal RNAs, and 32 transfer RNAs.

## GENOME ANNOUNCEMENT

*Borrelia hispanica* has been detected and isolated from specimens obtained in Northern Africa and Southern Europe, including Morocco, Spain, and Portugal (1–3). It is one of the classical pathogenic relapsing fever (RF) *Borrelia* infecting humans in Africa, along with *B. duttonii*, *B. recurrentis*, and *B. crocidurae*. Human cases of *B. hispanica* have been reported in 20.5% of patients in northwestern Morocco (4). Human infections with *B. hispanica* commonly lead to recurrent fever (4). In order to facilitate studies of *B. hispanica*, we sequenced the genome of *B. hispanica* strain CRI. The strain CRI was isolated from *Ornithodoros erraticus* ticks in Morocco. We succeeded in growing the bacteria in freshly prepared BSK II medium at 37°C, supplemented with 1.4% (wt/vol) gelatin and 10% (vol/vol) rabbit serum (5). Genomic DNA was extracted using a Wizard genomic DNA purification kit (Promega Biotech AB, Sweden).We propose *B. hispanica* strain CRI as a candidate type strain.

The complete genome sequence was determined by use of the Illumina HiSeq platform. Assembly of 12,427,984 reads with genomic coverage of 693-fold was performed using Abyss 1.3.4. Open reading frames (ORFs) were predicted using prodigal (6) and annotated by BLAST against the NCBI nonredundant database. Transfer RNAs and ribosomal RNAs were predicted using Aragorn and RNAmmer, respectively (7). To estimate the similarity at the genome level with African (RF) *Borrelia*, average nucleotide identity (ANI) was calculated (8).

The 1,783,846-bp genome of *B. hispanica* was almost completely collinear with other RF *Borrelia* species (9, 10). It consists of a 935,498-bp linear chromosome and 851,694-bp plasmids, with a G+C content of 28%. A total of 2,140 ORFs, 3 ribosomal RNAs, and 32 transfer RNAs were predicted. In the published genomes of African (RF) *Borrelia*, the phosphotransferase system (PTS) IIC chitibiose transporter protein is missing but it is present in *B. hispanica*. The ANI between the other African (RF) *Borrelia* species and *B. hispanica* is 96%. Availability of the four genomic sequence of the main pathogenic *Borrelia* in Africa will provide clues to the genome evolution and virulence factors of each species.

### Nucleotide sequence accession numbers.

This whole-genome shotgun project has been deposited at DDBJ/EMBL/GenBank under the accession no. AYOU00000000. The version described in this paper is version AYOU01000000.
